# Ocular adverse events of perioperative adjuvant docetaxel vs paclitaxel for breast cancer: propensity-score overlap-weighted analysis

**DOI:** 10.1007/s10549-025-07720-8

**Published:** 2025-05-13

**Authors:** Chikako Iwai, Atsushi Miyawaki, Takaaki Konishi, Akira Okada, Asahi Fujita, Taisuke Jo, Hideo Yasunaga

**Affiliations:** 1 Department of Clinical Epidemiology and Health Economics, School of Public Health, The University of Tokyo, 7-3-1 Hongo, Bunkyo-Ku, Tokyo, 113-0033 Japan; 2 Department of Health Services Research, Graduate School of Medicine, The University of Tokyo, Tokyo, Japan; 3 Department of Breast and Endocrine Surgery, Graduate School of Medicine, The University of Tokyo, Tokyo, Japan; 4 Department of Prevention of Diabetes and Lifestyle-Related Diseases, Graduate School of Medicine, The University of Tokyo, Tokyo, Japan; 5 Department of Ophthalmology, Graduate School of Medicine, The University of Tokyo, Tokyo, Japan

**Keywords:** Breast cancer, Cystoid macular edema, Epiphora, Optic neuropathy, Taxane

## Abstract

**Background:**

Taxanes are known to be associated with several ocular adverse events; however, large-scale data comparing the risk of ocular adverse events between the two commonly used taxanes, docetaxel (DTX) and paclitaxel (PTX), remain limited. Therefore, we aimed to compare the risks of epiphora, cystoid macular edema, and optic neuropathy between DTX- and PTX-based chemotherapy regimens.

**Methods:**

Using a nationwide claims database in Japan, we identified 6038 patients who initiated neoadjuvant or adjuvant taxane-based chemotherapy for breast cancer between April 2014 and November 2022. Data analysis was conducted in 2024. This study was conducted across multiple referral centers and community hospitals in Japan, providing a comprehensive view of taxane-based chemotherapy practices in diverse healthcare settings. Participants included 6038 patients diagnosed with breast cancer who initiated neoadjuvant or adjuvant taxane-based chemotherapy. Eligibility criteria included female patients aged 18 years or older. Participants were selected from a nationwide claims database using a consecutive sampling method. Patients who received DTX-based chemotherapy (DTX group) were compared with those who received PTX-based chemotherapy (PTX group). After adjusting for potential confounders using a propensity score-overlap weighting method, we estimated the incidence of the three ocular adverse events and hazard ratios (HRs) using Cox proportional hazards regression models.

**Results:**

Among the 6038 eligible patients, 3829 were in the DTX group and 2209 in the PTX group. The adjusted HR for epiphora in the DTX group was 1.69 [95% confidence interval (CI), 1.17 to 2.45] compared with the PTX group. For cystoid macular edema and optic neuropathy, the adjusted HRs were 0.74 (95% CI, 0.52 to 1.04) and 0.72 (0.47 to 1.11), respectively. The composite incidence of epiphora, cystoid macular edema, and optic neuropathy did not differ significantly between the DTX and PTX groups.

**Conclusion:**

Among patients receiving taxane-based chemotherapy for breast cancer, DTX was associated with a higher risk of epiphora compared with PTX. However, the incidence rates of cystoid macular edema and optic neuropathy did not differ significantly between the two groups. These findings offer valuable insights into the ocular adverse event profile of taxane-based chemotherapy in patients with breast cancer.

**Supplementary Information:**

The online version contains supplementary material available at 10.1007/s10549-025-07720-8.

## Introduction

Taxane-based agents, including docetaxel (DTX) and paclitaxel (PTX), are critical in improving prognosis for early-stage breast cancer [1–6]. These agents exert their antitumor effects by promoting microtubule polymerization and inhibiting cell division [7]. Despite their therapeutic efficacy, taxanes are associated with various adverse events, including myelosuppression, peripheral neuropathy, hypersensitivity reactions, and myalgia and arthralgia [8]. In addition, ocular adverse events associated with the use of DTX and PTX have been reported, with a primary focus on epiphora, cystoid macular edema, and optic neuropathy [9–15]. These ocular complications can significantly impact patients’ quality of life [16]. Epiphora can severely limit outdoor activities [12]. Furthermore, cystoid macular edema and optic neuropathy may lead to visual impairment [9–12].

However, previous studies reporting the occurrence of epiphora, cystoid macular edema, and optic neuropathy following the use of taxane-based agents are primarily limited to small case series [9–12, 14, 15, 17–21]. A recent study involving 18,219 patients found an increased risk of epiphora, cystoid macular edema, and optic neuropathy in women receiving taxane-based chemotherapy compared to those who received tamoxifens [13]. However, it remains unclear whether the incidence of ocular adverse events differs between the two commonly used taxanes, DTX and PTX. These agents may have distinct risks for ocular events due to differences in binding affinity to beta-tubulin, intracellular retention time, and pharmacokinetics [22, 23]. To our knowledge, no large-scale studies have compared the risk of ocular adverse events between DTX and PTX, except for a single-institution study [24].

This study aimed to compare the incidence of ocular adverse events, specifically epiphora, cystoid macular edema, and optic neuropathy, between DTX and PTX regimens in perioperative breast cancer treatment using a large administrative claims database in Japan.

## Methods

### Data source

This retrospective cohort study was conducted using the DeSC database (DeSC Healthcare, Inc., Tokyo, Japan), which contains claims data for approximately 12,500,000 insurance subscribers to several Japanese public health insurers [25, 26]. These include employees’ health insurance, non-employees’ health insurance, and the late elderly healthcare system for individuals aged 75 years or older. Mortality data were available for non-employees’ health insurance and the late elderly healthcare system. A previous study reported that the DeSC database closely reflects the age distribution of the Japanese population estimates [25].

Diagnoses were recorded using the International Classification of Diseases, 10th revision (ICD-10), and nationally standardized Japanese diagnosis codes. Drug specifications were documented according to the Anatomical Therapeutic Chemical classification system established by the World Health Organization. Information on each prescribed drug’s date, dose, and duration was available. Medical procedures were recorded using Japanese medical procedure codes.

The need for informed consent was waived because the patient database was anonymized. The study was approved by the Institutional Review Board of the University of Tokyo (approval number: 2021010NI, April 23, 2021).

### Patient selection

**Fig. 1:**
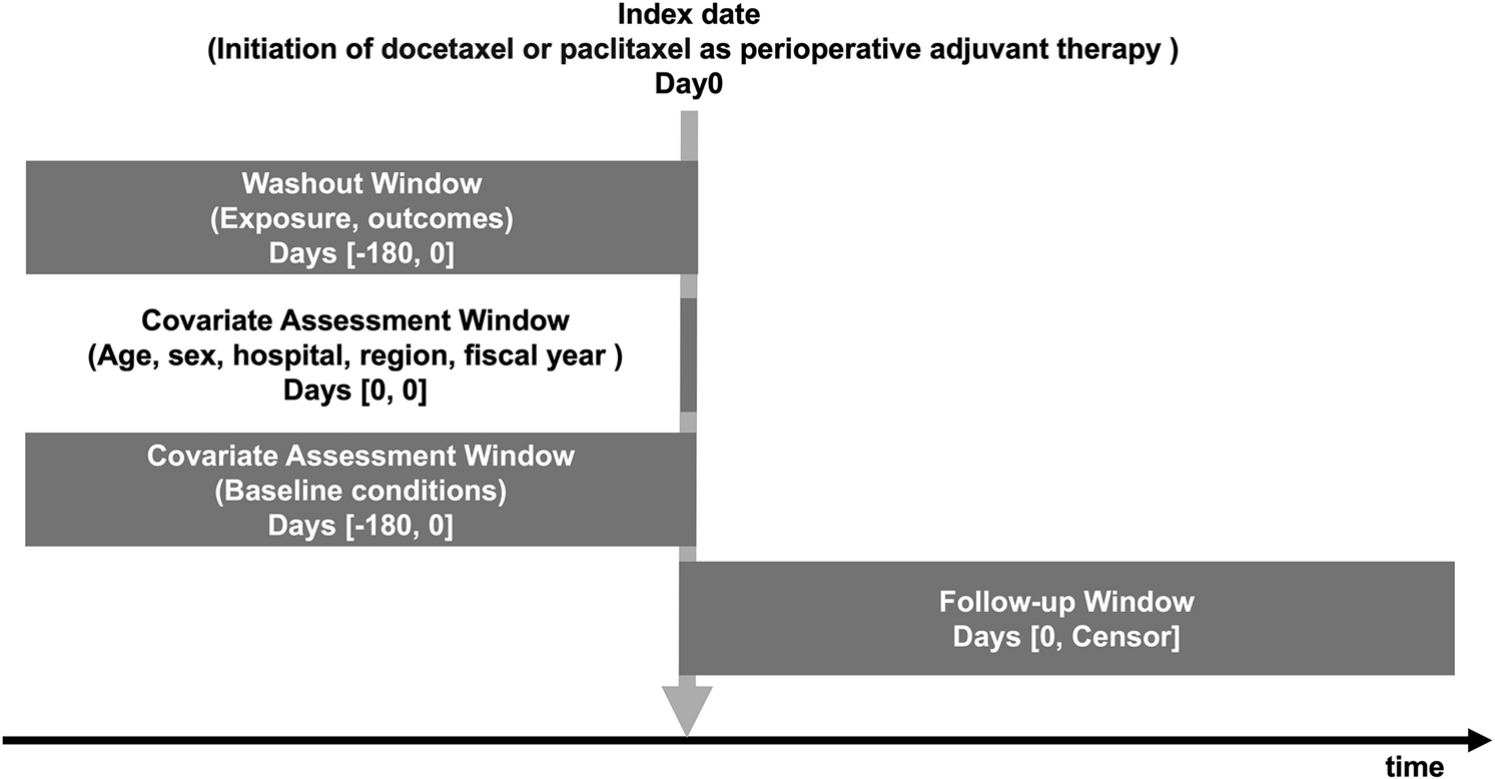
Study design

We identified patients with breast cancer (ICD-10 code, C50) aged 18 years or older who initiated DTX or PTX as perioperative adjuvant therapy between April 2014 and November 2022. The date of DTX or PTX initiation was defined as the index date. Patients were excluded if they joined the insurers included in the database within six months before the index date, as this would result in an insufficient lookback period or if they had a history of ocular outcomes (epiphora, cystoid macular edema, or optic neuropathy) within six months before the index date. Eligible patients were divided into the DTX and PTX groups according to the regimen. The study design is shown in Fig. 1.

### Outcomes measures

The primary outcomes were defined using the specific ICD-10 codes: epiphora (H20, H220, H221, H30, and H441), cystoid macular edema (H358), and optic neuropathy (H46, H47) [27]. The occurrence of each outcome was defined as the first appearance of an ophthalmic disease diagnosis. Follow-up was censored at the earlier of loss to follow-up (defined as loss of eligibility for health insurance) or the end of the study period (November 1, 2022).

### Adjustment variables

Covariates included age at the index date, sex, ophthalmic diseases, systemic comorbidities, other drug use, recent eye examination, hospital category, region, and year.

Age was categorized into ten groups: 18–39, 40–44, 45–49,50–54, 55–59, 60–64, 65–69, 70–74, 75–79, and ≥ 80 years. Ophthalmic diseases included: blepharitis, cataracts, dry eye, glaucoma (identified by antiglaucoma drug use) [28], pseudophakia, keratitis, ptosis, and uveitis. Regarding systemic comorbidities, we investigated autoimmune disorders, chronic kidney disease, coronary artery disease, diabetes mellitus, and hypertension using the relevant ICD-10 codes (Supplemental Table 1). Information on ophthalmic conditions, systemic comorbidities, and other drug use was obtained from diagnoses on the index date or within the six months prior to the index date. We counted the number of eye examinations within six months before the index date, categorizing it as zero or ≥ 1. The hospital category included academic hospitals or designated cancer care hospitals. The region was categorized into nine groups: Hokkaido/Tohoku, South Kanto, North Kanto/Koshin, Hokuriku, Tōkai, Kinki, Chugoku, Shikoku, and Kyushu/Okinawa.

### Statistical analysis

We applied the propensity-score overlap weighting method to balance covariates between the two groups. Overlap weighting minimizes the asymptotic variance of the nonparametric estimates of the weighted average treatment effect within each weight class [29–32]. Propensity scores were calculated using multivariable logistic regression with all the covariates mentioned above. We computed the weights based on the likelihood of each patient receiving the opposite treatment. Standardized differences were calculated to assess covariate balance between the two groups, with an absolute standardized difference of < 10% indicating negligible differences [33].

We compared Kaplan–Meier curves between the two groups using the log-rank test and calculated the incidence of outcomes (/10,000 person-years) after overlap weighting. Bootstrap methods with 1000 independent samples were used to calculate confidence intervals (CIs) and *P*-values for differences in incidence [34]. Hazard ratios (HRs) and their CIs were estimated using Cox proportional hazard models in the overlap-weighted cohorts to assess the associations of DTX use with outcomes. Robust variances were used to calculate CIs, accounting for sample weights [32, 35]. Additionally, analyses were stratified by age (< 65 and ≥ 65 years) to evaluate whether the associations between DTX and PTX use varied by patient age [36, 37].

### Secondary analyses

As a post-hoc analysis, we estimated HRs to evaluate the associations between DTX use and a composite outcome (the composite incidence of ocular epiphora, cystoid macular edema, and optic neuropathy) in the overlap-weighted cohorts using Cox proportional hazard models.

As a sensitivity analysis to assess the potential influence of unobserved confounders on the observed associations, we performed a falsification test [38], in which we examined the incidence of burn injuries (defined by ICD-10 codes T20–T32) as an alternative outcome. Since burn injuries are unrelated to the mechanism of action of taxanes, we hypothesized that the incidence of burn injuries would be similar between DTX and PTX if unobserved confounding was minimal.

All hypothesis tests were two-sided, with a statistical significance level set at 0.05. Statistical analyses were performed using Stata/SE 18.0 statistical software (StataCorp, College Station, TX, USA).

## Results

**Fig. 2:**
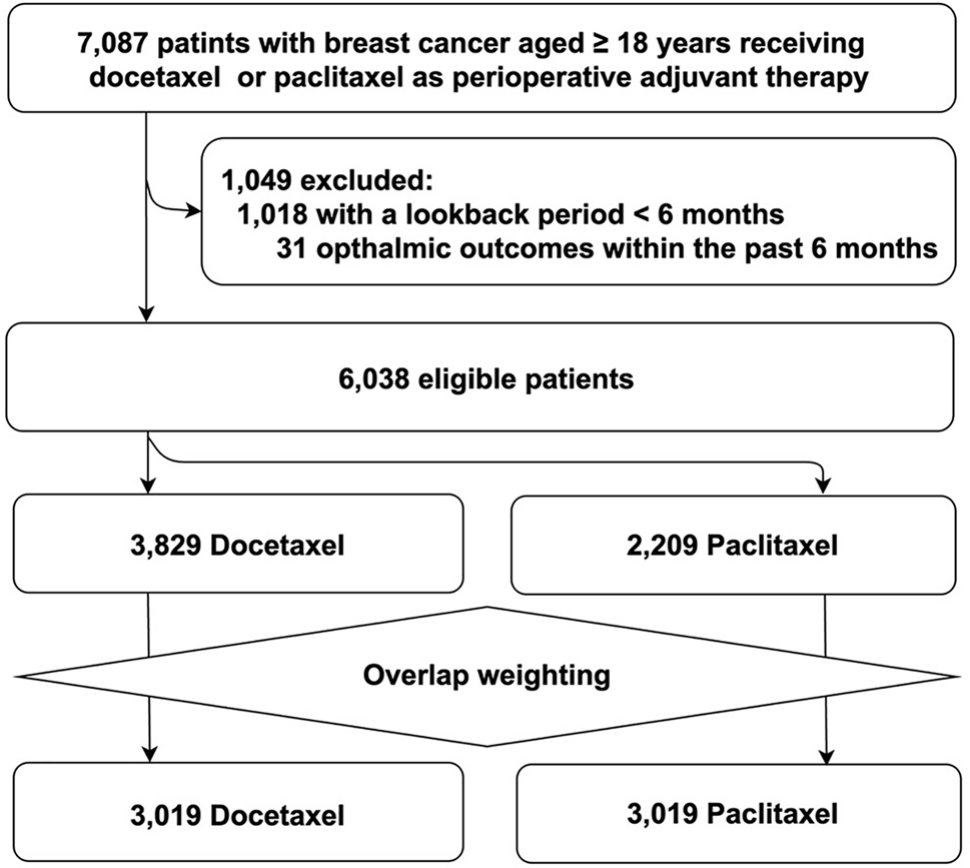
Flow diagram

We identified 7087 patients with breast cancer aged 18 years or older who received DTX or PTX as perioperative adjuvant therapy. After excluding 1052 patients based on the exclusion criteria (Fig. 2), 6038 patients remained eligible, with 3829 in the DTX group and 2209 in the PTX group.

Table 1 presents the baseline characteristics before and after applying propensity score-overlap weighting for patients treated with DTX or PTX. Before weighting, the DTX and PTX groups showed differences in age and regional distribution. After weighting, each group comprised 3019 patients, and the covariates were completely balanced (i.e., all absolute standardized differences were zero). The median follow-up was 851 days (interquartile range, 393 to 1310) in the DTX group and 731 days (365 to 1247) in the PTX group. The median age was 65 years (interquartile range, 54 to 70).

**Table 1:** Demographic and clinical characteristics of patients

	Before overlap weighting					After overlap weighting				
	Docetaxel		Paclitaxel		ASD^*^	Docetaxel		Paclitaxel		ASD^*^
	n = 3829		n = 2209		(%)	n = 3019		n = 3019		(%)
Age category, years										
18–39	112	(2.9)	66	(3.0)	0.4	94	(3.1)	94	(3.1)	0.0
40–44	201	(5.2)	103	(4.7)	2.7	153	(5.1)	153	(5.1)	0.0
45–49	382	(10)	139	(6.3)	13.5	230	(7.6)	230	(7.6)	0.0
50–54	364	(9.5)	170	(7.7)	6.5	261	(8.6)	261	(8.6)	0.0
55–59	347	(9.1)	168	(7.6)	5.3	256	(8.5)	256	(8.5)	0.0
60–64	559	(15)	247	(11)	10.2	388	(13)	388	(13)	0.0
65–69	973	(25)	464	(21)	10.5	708	(23)	708	(23)	0.0
70–74	579	(15)	373	(17)	4.8	513	(17)	513	(17)	0.0
75–79	258	(6.7)	321	(15)	25.5	326	(11)	326	(11)	0.0
≥ 80	54	(1.4)	158	(7.2)	28.7	90	(3.0)	90	(3.0)	0.0
Sex (female)	3813	(99.6)	2203	(99.7)	2.5	3010	(99.7)	3010	(99.7)	0.0
Pre-existing ophthalmic diseases										
Blepharitis	10	(0.5)	3	(0.1)	5.9	5	(0.2)	5	(0.2)	0.0
Cataract	95	(2.5)	76	(3.4)	5.7	93	(3.1)	93	(3.1)	0.0
Dry eye	79	(2.1)	55	(2.5)	2.9	73	(2.4)	73	(2.4)	0.0
Glaucoma	17	(0.8)	16	(0.7)	0.5	19	(0.6)	19	(0.6)	0.0
Pseudophakia	22	(1.0)	15	(0.7)	3.5	19	(2.0)	19	(2.0)	0.0
Keratitis	73	(1.9)	48	(2.2)	1.9	61	(0.1)	61	(0.1)	0.0
Ptosis	2	(0.05)	1	(0.03)	1.3	2	(0.07)	2	(0.07)	0.0
Uveitis	7	(0.3)	7	(0.3)	0.0	7	(0.2)	7	(0.2)	0.0
Systemic comorbidities										
Autoimmune disorder	18	(0.8)	14	(0.6)	2.1	16	(0.5)	16	(0.5)	0.0
Chronic kidney diseases	12	(0.5)	12	(0.5)	0.0	13	(0.4)	13	(0.4)	0.0
Coronary artery diseases	83	(2.2)	50	(2.3)	0.7	67	(2.2)	67	(2.2)	0.0
Diabetes mellitus	215	(5.6)	113	(5.1)	2.2	157	(5.2)	157	(5.2)	0.0
Hypertension	185	(4.8)	127	(5.7)	4.1	163	(0.4)	163	(0.4)	0.0
Medications										
Insulin	133	(3.5)	108	(4.9)	7.1	129	(4.3)	129	(4.3)	0.0
Oral antidiabetic agent	63	(1.6)	37	(1.7)	0.2	47	(1.6)	47	(1.6)	0.0
Oral corticosteroids	2790	(73)	1580	(72)	3.0	2183	(72)	2183	(72)	0.0
Tamoxifen	73	(1.9)	72	(3.3)	8.5	31	(1.0)	31	(1.0)	0.0
Recent eye examination ≥ 1	200	(5.2)	137	(6.2)	4.2	171	(5.7)	171	(5.7)	0.0
Hospital category^**^	1680	(44)	947	(43)	2.0	1302	(43)	1302	(43)	0.0
Region										
Hokkaido/Tohoku	63	(1.6)	21	(1.0)	6.1	37	(1.2)	37	(1.2)	0.0
South Kanto	639	(17)	341	(15)	3.4	507	(17)	507	(17)	0.0
North Kanto/Koshin	834	(22)	243	(11)	29.4	422	(14)	422	(14)	0.0
Hokuriku	86	(2.2)	42	(1.9)	2.4	65	(2.2)	65	(2.2)	0.0
Tōkai	1349	(35)	811	(37)	3.1	1129	(37)	1129	(37)	0.0
Kinki	352	(9.2)	407	(18)	27.0	405	(13)	405	(13)	0.0
Chugoku	212	(5.5)	114	(5.2)	1.7	172	(5.7)	172	(5.7)	0.0
Shikoku	230	(6.0)	187	(8.5)	9.5	228	(7.6)	228	(7.6)	0.0
Kyushu/Okinawa	63	(1.6)	43	(1.9)	2.3	54	(1.8)	54	(1.8)	0.0
Year										
2014	37	(1.0)	25	(1.1)	1.6	34	(1.1)	34	(1.1)	0.0
2015	285	(7.4)	154	(7.0)	1.8	226	(7.5)	226	(7.5)	0.0
2016	377	(9.8)	179	(8.1)	6.1	264	(8.7)	264	(8.7)	0.0
2017	485	(13)	249	(11)	4.3	360	(12)	360	(12)	0.0
2018	577	(15)	389	(18)	6.9	490	(16)	490	(16)	0.0
2019	697	(18)	404	(18)	0.2	543	(18)	543	(18)	0.0
2020	672	(18)	426	(19)	4.5	567	(19)	567	(19)	0.0
2021	605	(16)	321	(15)	3.5	450	(15)	450	(15)	0.0
2022	94	(2.5)	62	(2.8)	2.2	85	(2.8)	85	(2.8)	0.0

Data are presented as n (%)

*ASD* absolute standardized difference

*An ASD of < 10% denotes a negligible difference between the two groups

**Hospital category includes academic hospitals and designated cancer care hospital

Table 2 presents the incidences of the outcomes after overlap weighting. The incidence of epiphora differed significantly between the two groups [128 vs. 77 cases per 10,000 person-years; difference, 51 (95% CI 1 to 92) cases per 10,000 person-years]. The incidences of cystoid macular edema and optic neuropathy were 87 vs. 118 per 10,000 person-years and 55 vs. 75 per 10,000 person-years, respectively, with no significant differences between the two groups. In the stratified analysis, there were 2858 individuals aged < 65 years [median age, 53 years (interquartile range, 47 to 60 years)] and 3180 individuals aged ≥ 65 years [median age, 70 years (68 to 74 years)]. No significant differences were observed between the two groups for any of the outcomes, regardless of age category.

**Table 2:** Incidence of outcomes after overlap weighting

	Incidence (/10000 person-years)			*P-*value
	Docetaxel	Paclitaxel	Difference (95% CI^*^)	
Epiphora				
Overall	128	77	− 51 (1 to 93)	0.044
< 65 years old	62	33	− 29 (− 18 to 65)	0.27
≥ 65 years old	194	121	− 73 (7 to 150)	0.075
Cystoid macular edema				
Overall	87	118	− 51 (− 68 to 26)	0.38
< 65 years old	29	55	− 26 (− 60 to 32)	0.54
≥ 65 years old	147	180	− 33 (− 107 to 62)	0.60
Optic neuropathy				
Overall	55	75	− 51 (− 63 to 136)	0.19
< 65 years old	31	56	− 25 (− 74 to 17)	0.22
≥ 65 years old	77	95	− 18 (− 81 to 35)	0.43

*CI* confidence interval

*Using bootstrap methods of 1000 independent samples, we calculated confidence intervals for the difference in incidence and p-values

**Fig. 3:**
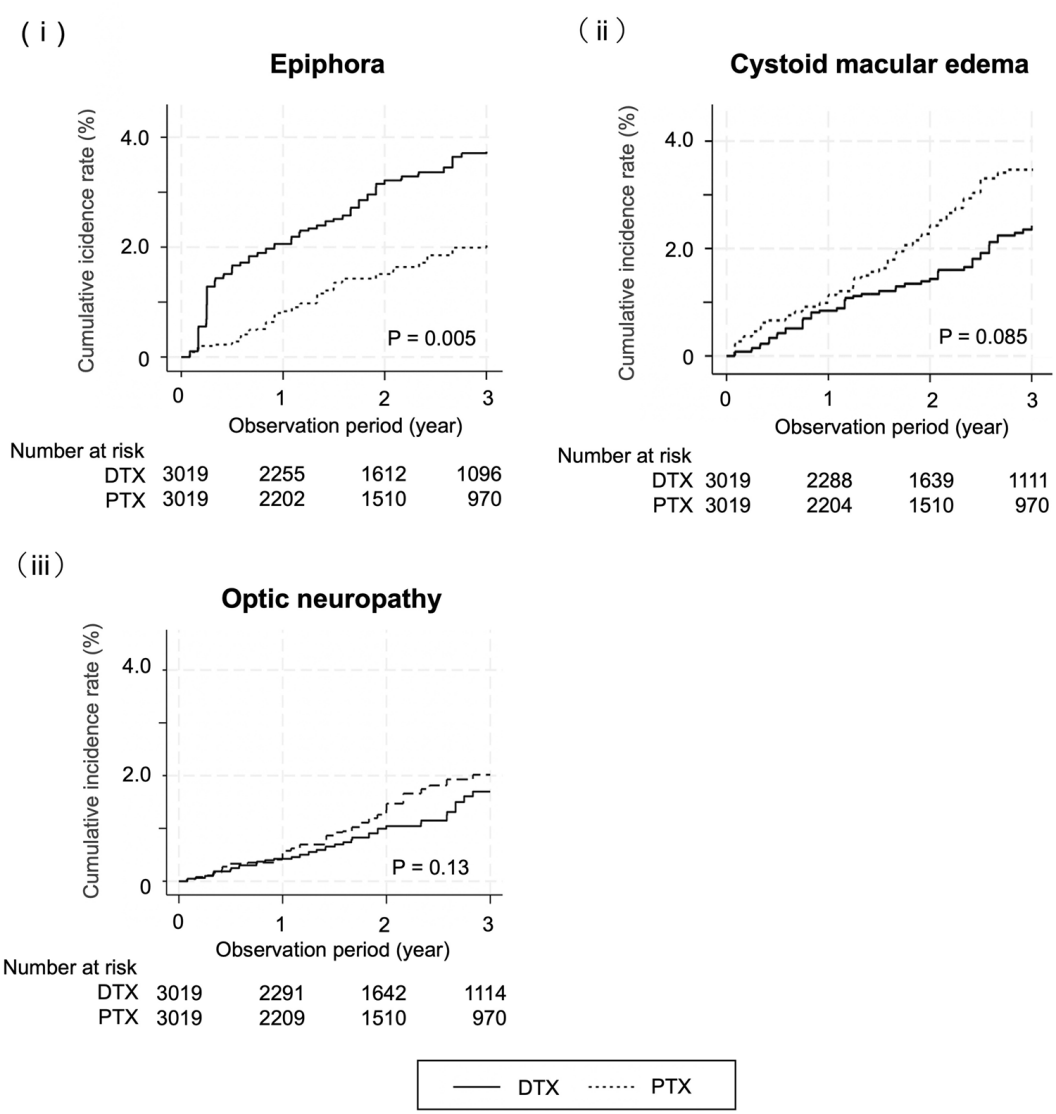
Cumulative probability for ocular adverse events. *DTX* docetaxel, *PTX* paclitaxel

Figure 3 shows cumulative probabilities for ocular adverse events. Epiphora occurred more frequently in the DTX group compared with the PTX group (log-rank test, *P* = 0.005).

**Fig. 4:**
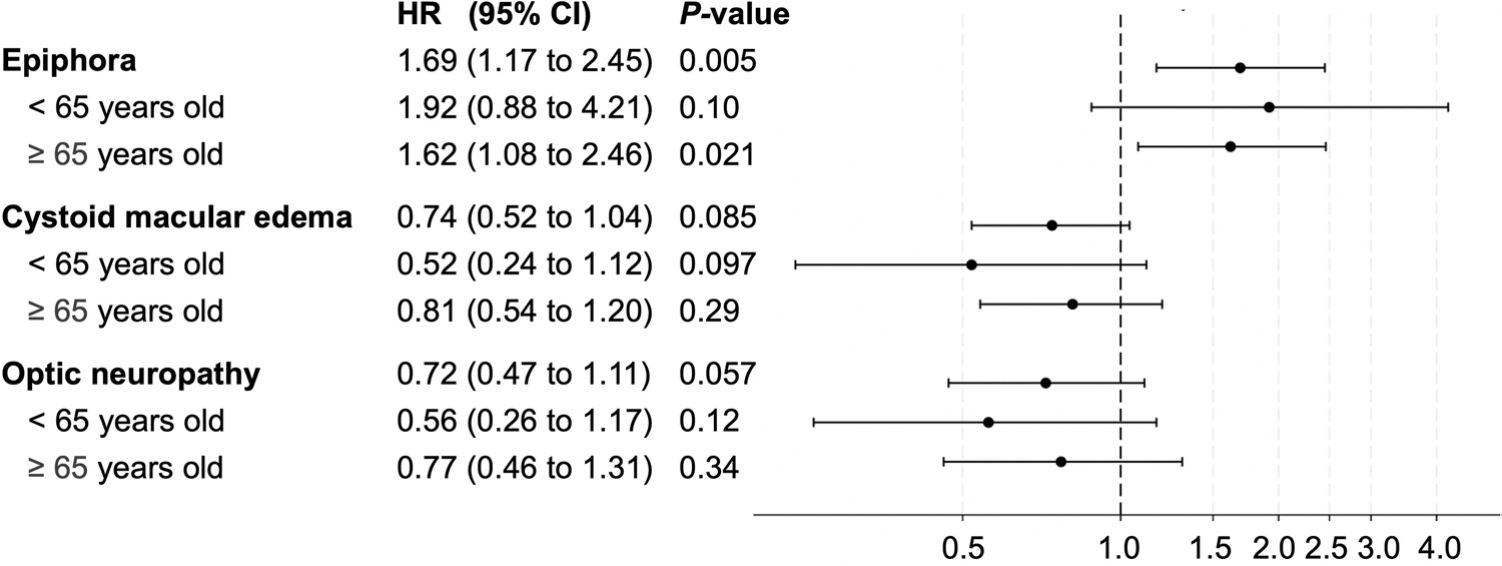
Adjusted hazard ratios and 95% CI for outcomes after overlap weighting. *HR* hazard ratio, *CI* confidence interva

Figure 4 shows the adjusted HRs for the outcomes after overlap weighting. The HR for epiphora in the DTX group was significant at 1.69 (95% CI, 1.17 to 2.45). The HRs for cystoid macular edema and optic neuropathy were 0.74 (95% CI, 0.52 to 1.04) and 0.72 (95% CI, 0.47 to 1.01), respectively. In the age-stratified analysis, the point estimates were consistent with the main analysis, and there were no significant differences for any outcomes within age subgroups.

Post-hoc analysis showed that the composite outcome was comparable between the two groups [HR 1.02 (95% CI, 0.82 to 1.27)] (Supplemental Table 2). The falsification analysis showed no association between the two groups [HR 1.04 (95% CI, 0.79 to 1.38)].

## Discussion

This large-scale retrospective analysis of real-world clinical data provides valuable insight into the ocular adverse events profiles of DTX and PTX by comparing the risk of these events (epiphora, cystoid macular edema, and optic neuropathy) following DTX and PTX administration in perioperative adjuvant therapy for patients with breast cancer. In the post-hoc analysis, the composite outcome of ocular adverse events was comparable between the two groups. However, individual outcomes showed significant differences. Specifically, the DTX group had a significantly higher risk of epiphora than the PTX group. The DTX group had relatively lower risks of cystoid macular edema and optic neuropathy compared with the PTX group, but the differences were not significant.

Intravenously administered taxane agents enter the tear fluid from the plasma and can induce stenosis of the lacrimal ducts due to inflammation and scar formation [17, 39]. Additionally, dry eye due to meibomian gland dysfunction could result in reflexive epiphora without canalicular stenosis [40, 41]. In the current study, we confirmed a high risk of epiphora following DTX use. This trend is consistent with findings from previous studies. Epiphora and nasolacrimal duct stenosis caused by DTX have been reported to be more frequent than those caused by PTX [14, 17–21]; for example, the U.S. Food and Drug Administration Adverse Event Reporting System showed that the proportional reporting ratio was 2.47 (95% CI, 2.03 to 3.02) [42]. The differences in risk between DTX and PTX may be attributed to differences in their binding affinity to β-tubulin, intracellular retention time, and pharmacokinetics [22, 23, 43]. Furthermore, additives used to enhance solubility may also play a role. Polysorbate 80 in docetaxel can induce inflammation in mucous membranes [44], whereas polyethylene castor oil in paclitaxel has anti-inflammatory properties [45]. These additives might influence the inflammation in the lacrimal ducts caused by taxanes migrating into the tear fluid. Epiphora has been reported to significantly affect daily life, particularly by impairing outdoor activities and interpersonal relationships [12]. These effects may further increase the stress associated with cancer treatment [46, 47]. Therefore, healthcare providers and patients need to recognize the risk of DTX-induced epiphora for early symptom recognition and appropriate ophthalmological evaluation. Depending on the severity of symptoms, discontinuation of DTX and necessary treatments (e.g., artificial tears, punctual plugs, surgery) could alleviate epiphora discomfort and improve patients’ quality of life [40].

PTX has been reported to be more likely associated with cystoid macular edema than DTX [48]. The current study showed that the use of DTX, compared with PTX, was associated with lower hazard ratios, but the difference was not significant, and therefore no definitive conclusion could be drawn. Several hypotheses have been proposed regarding the pathogenesis of taxane-induced cystoid macular edema. One theory is that the toxicity to Müller cells disrupts the blood-retinal barrier [49], and another suggests that taxanes impair the microtubule function of retinal pigment epithelium [50].

A recent study reported that taxane use increased the risk of optic neuropathy compared with tamoxifen [13]. The mechanisms underlying optic neuropathy may include optic nerve vascular ischemia, neurotoxicity due to axonal damage, and electrophysiological abnormalities [51, 52]. In the current study, the use of DTX, compared with PTX, showed a tendency toward a lower risk of optic neuropathy, although the difference was not significant.

Age-stratified analysis revealed that patterns of ocular adverse events were similar between patients aged under 65 and those aged 65 years and older. Although older patients represent a vulnerable population due to age-related changes in tear production and lacrimal anatomy [53], younger patients require similar attention, particularly regarding epiphora.

The current study provides new evidence on the risk of ocular adverse events between DTX and PTX in perioperative breast cancer treatment. Previous studies have focused on ocular adverse events associated with taxanes in general [13] or were based on limited case series [9–12, 14, 15, 17–21]. The present study identifies the distinct ocular risk profiles of DTX and PTX. The findings offer valuable guidance for healthcare providers to consider individual risks and implement appropriate measures for managing patient symptoms.

These findings emphasize the importance of individualized patient management in taxane-based chemotherapy. Early recognition of DTX-induced epiphora, coupled with appropriate ophthalmological evaluation, could alleviate symptoms and improve quality of life. Management strategies such as artificial tears, punctual plugs, or surgical interventions should be considered for severe cases. For PTX users, heightened vigilance for cystoid macular edema may be warranted, with regular retinal evaluations to mitigate potential vision impairment.

This study has several limitations. First, the severity of ophthalmic outcomes and their degree of impact on vision were not captured, which may limit the clinical applicability of our findings. The necessary treatment for ophthalmic diseases depends on their symptoms and severity; therefore, this information may be useful for clinical decision-making. Second, the current study focused on perioperative treatment for breast cancer and the findings may not be applicable to the treatment of advanced or recurrent breast cancer, as differences in drug dosage, treatment duration, and patients’ general condition could alter the risk profile for ocular adverse events [54]. Third, breast cancer staging, histopathological results, hormone receptor status, and human epidermal growth factor receptor 2 status may influence both treatment selection and ocular outcomes. However, data on these factors were not available in the current study, leading to potential residual confounding.

## Conclusion

Among patients who initiated taxane-based chemotherapy for breast cancer, the DTX group showed a higher risk of epiphora than the PTX group. In contrast, the risks of cystoid macular edema and optic neuropathy tended to be lower in the DTX group compared with the PTX group, but the differences were not statistically significant. These results suggest differences in the risk profiles of specific ocular adverse events between DTX and PTX, providing important information that should be considered for patient monitoring and overall safety assessments during taxane-based chemotherapy in patients with breast cancer.

## Supplementary Information

Below is the link to the electronic supplementary material.

Supplementary file1 (DOCX 26 KB)

## Data Availability

The data analyzed during this study are not publicly available due to contracts with the hospitals providing data to the database. Further inquiries regarding the data can be directed to the corresponding author.

## Abbreviations

CI: Confidence interval
DTX: Docetaxel
HR: Hazard ratio
ICD-10: International classification of diseases, 10 th revision
PTX: Paclitaxel
