# Supplementary material for: Ocular adverse events of perioperative adjuvant docetaxel vs paclitaxel for breast cancer: propensity-score overlap-weighted analysis
Source: Breast Cancer Res Treat. 2025 May 13;212(1):173–82. doi: 10.1007/s10549-025-07720-8 (PMC12086119; doi:10.1007/s10549-025-07720-8)
Supplement: Supplementary file 1 — Supplementary file1 (DOCX 26 KB) [file 10549_2025_7720_MOESM1_ESM.docx]

**Ocular Adverse Events of Perioperative Adjuvant Docetaxel vs Paclitaxel for Breast Cancer**

**Chikako Iwai, Atsushi Miyawaki, Takaaki Konishi, Akira Okada, Asahi Fujita, Taisuke Jo, Hideo Yasunaga**

**Supplemental Table 1** Definitions of ICD-10 Codes

**Supplemental Table 2** Adjusted hazard ratio for composite outcome in the post hoc analysis after overlap weighting

**Supplemental Table 1** Definitions of ICD-10 Codes

| **Diseases** | **ICD-10 Codes** |
| --- | --- |
| Breast cancer | C50 |
| ***Ophthalmic outcomes*** | I26.0, I26.9, I80.1, I80.2, I80.3, I80.8, I80.9, I82.8 |
| Epiphora  Cystoid macular edema | H045, H042 |
|  | H358 |
| Optic neuropathy | H46, H47 |
| ***Ophthalmic diseases*** |  |
| Blepharitis | H01.1 |
| Cataract | H25, H260, H262, H263, H264, H268, H269 |
| Dry eye | H04.1 |
| Intraocular lens insertion | Z961 |
| Keratitis | H16 |
| Ptosis | H024 |
| Uveitis | H20, H220, H221, H30, H441 |
| ***Systemic comorbidities*** |  |
| Autoimmune diseases | M05.0, M05.2, M05.3, M05.8, M05.9, M06, M30, M31, M32, M33, M34, M35, M36 |
| Chronic kidney diseases | N18 |
| Coronary artery diseases | I20.0, I20.1, I20.8, I20.9, I21.0, I21.1, I21.2, I21.3, I21.4, I21.9 |
| Diabetes | E10.2–E10.5, E10.7, E11.2–E11.5, E11.7, E12.2–E12.5, E12.7, E13.2–E13.5, E13.7, |
|  | E14.2–E14.5, E14.7 |
|  | E10.0, E10.1, E10.6, E10.8, E10.9, E11.0, E11.1, E11.6, E11.8, E11.9, E12.0, E12.1, |
|  | E12.6, E12.8, E12.9, E13.0, E13.1, E13.6, E13.8, E13.9, E14.0, E14.1, E14.6, E14.8 E14.9 |
| Hypertension | I10–15 |

ICD-10: International Classification of Diseases, Tenth Revision

**Supplemental Table 2** Adjusted hazard ratio for composite outcome in the post hoc analysis after overlap weighting

|  | **HR** | **95% CI** | ***P-*value** |
| --- | --- | --- | --- |
| **Composite outcome*** |  |  |  |
| Overall | 1.02 | 0.82 to 1.27 | 0.88 |
| < 65 years old | 0.86 | 0.56 to 1.32 | 0.50 |
| ≥ 65 years old | 1.07 | 0.83 to 1.38 | 0.59 |

HR, hazard ratio; CI, confidence interval
*Composite outcome, at least one of the following: epiphora, cystoid macular edema, or optic neuropathy.
